# Top management team career experience heterogeneity, digital transformation, and the corporate green innovation: a moderated mediation analysis

**DOI:** 10.3389/fpsyg.2023.1276812

**Published:** 2023-10-26

**Authors:** Daquan Gao, Songsong Li, Chang Guo

**Affiliations:** ^1^School of Management, Harbin Institute of Technology, Harbin, China; ^2^Business Division, School of Fashion and Textiles, Hong Kong Polytechnic University, Kowloon, Hong Kong SAR, China; ^3^Faculty of Arts and Social Sciences, Hong Kong Chu Hai College, Tsuen Wan, Hong Kong SAR, China

**Keywords:** top management team, digital transformation, green innovation, industry-academia-research collaboration, state owned enterprises, Yangtze River Economic Belt

## Abstract

**Introduction:**

Drawing upon upper echelon theory and the resource-based view, this study employs a moderated mediation model to investigate the moderating role and underlying mechanisms of digital transformation in the influence of top management teams (TMT) on corporate green innovation.

**Methods:**

Our analysis of panel data from 19,155 Chinese A-share listed companies (2011–2020) demonstrates that TMT career experience heterogeneity has a positive effect on green innovation, a relationship that is further strengthened by digital transformation.

**Results:**

This study shows the role of digital transformation in amplifying the effects of TMT diversity on green innovation and the crucial role of industry-academia-research collaboration as a mediator. Heterogeneity analysis highlights that non-state-owned enterprises (non-SOEs) show more agility than state-owned enterprises (SOEs) in leveraging heterogeneous TMT to drive green innovation. Conversely, green innovation in SOEs benefits more from digital transformation, which includes both its direct and indirect effects of digital transformation. Enterprises located in non-Yangtze River Economic Belt regions benefit more from digital transformation, demonstrating the importance of a balanced distribution of digital resources.

**Discussion:**

This study provides novel insights into leveraging inclusive leadership and digital capabilities to enhance ecological sustainability. This study underscores the potential of diversified TMTs and digitalization technology integration to catalyze green innovation, which is critical for environmentally responsible transformation.

## 1. Introduction

The increasing severity of environmental challenges has galvanized global call-to-action, prompting governments, societies, and businesses to re-evaluate their impact on the environment (Ahmad et al., 2018). This shift in societal perceptions has catalyzed governments to enforce stricter environmental regulations (Chen H. et al., 2022), compelling businesses to adopt more sustainable practices and green innovation (Zheng M. et al., 2022). Simultaneously, mounting societal concern over environmental degradation has increased the pressure on corporations to act responsibly, leading to heightened demand for corporate accountability (Richard et al., 2009). Consequently, an increased focus on green innovation, which emphasizes the development and implementation of environmentally friendly technologies and processes, has surfaced as a potential solution (Gao et al., 2023).

Enterprises driven by regulatory pressures and societal expectations are increasingly integrating green innovation into strategic planning (He et al., 2023). Upper Echelon Theory suggests that the values, experiences, and personalities of Top Management Teams (TMTs) significantly shape strategic decision-making and are mirrored in the resulting strategic choices (Hambrick, 2007; Hewa Heenipellage et al., 2022). Given the integral role of TMTs in shaping strategic direction (Samimi et al., 2022) and overcoming resistance to change (Richard et al., 2019), it is essential to understand how TMTs impact green innovation. While current research largely examines individual executive characteristics such as CEO age (E-Vahdati and Binesh, 2021), gender (Javed et al., 2023), and education (Zhao et al., 2021) on green innovation, less attention has been given to collective TMT characteristics such as overseas experience (Meng et al., 2022). While research is shifting toward examining TMTs and green innovation (Hambrick, 2007), there remains a theoretical and empirical gap concerning the effects of TMT heterogeneity on green innovation. Most notably, the study of TMT career experience heterogeneity is of paramount importance. This study argues that diversity in TMT career experiences creates an environment that nurtures green innovation, stemming from a wealth of diverse ideas and perspectives (Heyden et al., 2017). This diversity within TMTs could potentially lead to more innovative and effective green strategies; thus, the importance of studying this diversity cannot be overstated. By examining the role of TMT heterogeneity in driving green innovation, this study contributes to the literature on upper echelons theory and the resource-based view of firms.

Digital transformation is a significant catalyst for organizational change, disrupting traditional business models and fostering innovation, including green innovation (Niu et al., 2023). From the Resource-Based View (RBV), this study posits that the heterogeneity of Top Management Teams (TMT), a unique and valuable resource, can encourage green innovation by incorporating diverse knowledge, skills, and perspectives (Samimi et al., 2022). However, the benefits of TMT heterogeneity are maximized when complemented by digital transformation, which can enhance diversity-driven advantages through improved communication, collaboration, and knowledge-sharing (Ahmad and Karim, 2019). This study introduces a mediating factor: cooperation between industry, academia, and research entities. This collaboration, which is significantly enhanced through digital transformation, can stimulate knowledge sharing and joint innovation, both of which are critical for green innovation (He and Liu, 2023). Therefore, we propose a Moderated Mediation Model in which digital transformation moderates the impact of TMT heterogeneity on green innovation, with the effect mediated via industry-academia-research cooperation (Nájera-Sánchez et al., 2022). This model provides a more nuanced understanding of how digital transformation and TMT heterogeneity jointly influence green innovation.

What is the impact of top management team (TMT) heterogeneity and digital transformation on corporate green innovation? To answer this question, this study proposes a Moderated Mediation Model that delivers substantial theoretical and practical contributions. From a theoretical perspective, this research expands the application of upper echelons theory and the resource-based view to green innovation domains by elucidating how diverse TMTs can drive eco-innovation as unique strategic resources. The integrated model further enriches the theoretical understanding of how team diversity characteristics translate into sustainability strategies. This novel application extends the boundaries of predominant management theories into an increasingly important context. Practically, the study provides actionable guidance to managers and policymakers. The results underline the value of cultivating TMT diversity, investing in digital capabilities, and actively fostering external collaboration for green innovation. For companies, this points to progressive team composition, strategic digital resource allocation, and partnership orientation to leverage innovation opportunities. For policymakers, a regulatory environment facilitating digitization and collaboration is essential to unlock green progress. Overall, the integrated theoretical model and empirical findings contribute significant insights into how organizations can leverage inclusive leadership, technological integration, and strategic partnerships to achieve ecological sustainability. This fuses management theory with practice, delivering a research exemplar to catalyze future studies.

## 2. Literature review and hypothesis development

### 2.1. Upper echelon theory and corporate green innovation

The Upper Echelons Theory, proposed by Hambrick and Mason (1984), posits that business executives’ distinct traits derived from their past educational and professional experiences shape their psychological structures, including attentional tendencies, cognitive abilities, and values. Once formed, these traits equip decision makers with problem-solving approaches and a repository of past solutions applicable to current challenges (Zhang and Greve, 2018). This process results in behavioral tendencies to repeat familiar actions, and cognitive tendencies to categorize and consider problems in familiar ways. Consequently, decision makers favor decisions that resonate with their experiences. Recently, the application of this theory to corporate green innovation has gained traction following a shift from a traditional focus on internal and external factors to the significant role of corporate top management teams (Hambrick, 2007). The bounded rationality of humans suggests that because of the complexity of corporate environments, it is not practical for managers to possess exhaustive knowledge of all corporate operations. Instead, top managers’ personalized interpretations of the issues their firms encounter lead to diversified corporate strategies (Talke et al., 2010; Puranam et al., 2015). As a result, top management teams wield substantial influence over strategic directions, resource allocation, and investments, thereby driving change and innovation (Carpenter et al., 2004). This perspective recognizes the collective impact of the top management team on green innovation and sustainability practices, emphasizing the importance of their unique traits and experiences in shaping strategic decisions.

Current articles on corporate executives and green innovation can be categorized into four groups. The first examines the impact of corporate CEOs’ innate characteristics on green innovation, such as CEO age (E-Vahdati and Binesh, 2021) and gender (Javed et al., 2023). The second category focuses on the impact of corporate CEOs’ acquired characteristics on green innovation such as educational experience (Zhao et al., 2021), overseas experience (Quan et al., 2021), hometown identification (Ren et al., 2021), trustworthiness (Ullah et al., 2023), hubris (Arena et al., 2018), and managerial myopia (Liu, 2022). The third category examines the impact of the innate characteristics of top management teams (TMT) on green innovation, such as age (Guo and Ma, 2022) and bio-demographic fault lines (Ma et al., 2021). The fourth category investigates the impact of acquired TMT characteristics on green innovation, such as overseas experience (Meng et al., 2022) and academic experience (Zhou et al., 2021).

### 2.2. Resource-based view

The Resource-Based View (RBV) is a prominent theory in strategic management that elucidates how firms acquire and sustain competitive advantages through resource and capability heterogeneity (Barney et al., 2011). Resources are defined as any asset that can yield value for a firm, and when paired with capabilities—the capacity of a firm to leverage these resources—they underpin sustainable competitive advantage. This advantage is secured by the difficulty competitor’s face in imitating or substituting these unique resources and capabilities (Peteraf, 1993).

This theory can complement the Upper Echelon Theory (UET), which asserts that a firm’s outcomes echo the characteristics of its top management team (TMT; Hambrick and Mason, 1984). A significant limitation of UET is the “black box” problem, which leaves the mechanics of decision making largely unexplained (Neely et al., 2020). By explaining why certain strategic decisions lead to competitive advantage and superior performance, the RBV can help decode this “black box” (Kraaijenbrink et al., 2009). Notably, heterogeneity in the career experiences of the TMT can be a distinctive and valuable resource that potentially stimulates green innovation (Table 1).

**Table 1:** Research on upper echelon theory and green innovation.

Type	Research objectives	Authors
CEO innate characteristics	Female CEO	Javed et al. (2023)
	CEO age	E-Vahdati and Binesh (2021)
CEO acquired characteristics	CEO Hubris	Arena et al. (2018)
	CEO education experience	Zhou et al. (2021)
	CEO foreign experience	Quan et al. (2021)
	CEO hometown identity	Ren et al. (2021)
	CEO political connections	Huang et al. (2021)
	CEO trustworthiness	Ullah et al. (2023)
	CEO managerial myopia	Liu (2022)
TMT innate characteristics	TMT age	Guo and Ma (2022)
	TMT bio-demographic fault lines	Ma et al. (2021)
TMT acquired characteristics	TMT ethical leadership	Xuecheng and Iqbal (2022)
	TMT environmental awareness	Sun and Sun (2021); Wang et al. (2022)
	TMT oversea experience	Chen W. et al. (2022); Meng et al. (2022)
	TMT academic experience	Zhao et al. (2021)
	TMT emotional framing	Fang and Zhang (2021)

The digital transformation of a business, that is, the employment of digital technologies to modify business processes or models, is crucial in enabling companies to develop new resources and capabilities (Vial, 2019; Verhoef et al., 2021). One such capability bolstered by digital transformation is external collaboration, which forms value-creating alliances with other organizations (He et al., 2023). In the context of green innovation, industry-academia-research cooperation is introduced as an essential mediator and unique resource. This cooperation, significantly amplified by digital transformation, fosters improved knowledge sharing and joint innovation, which are critical factors in green innovation (Albort-Morant et al., 2017; Singh et al., 2020).

**Figure 1:**
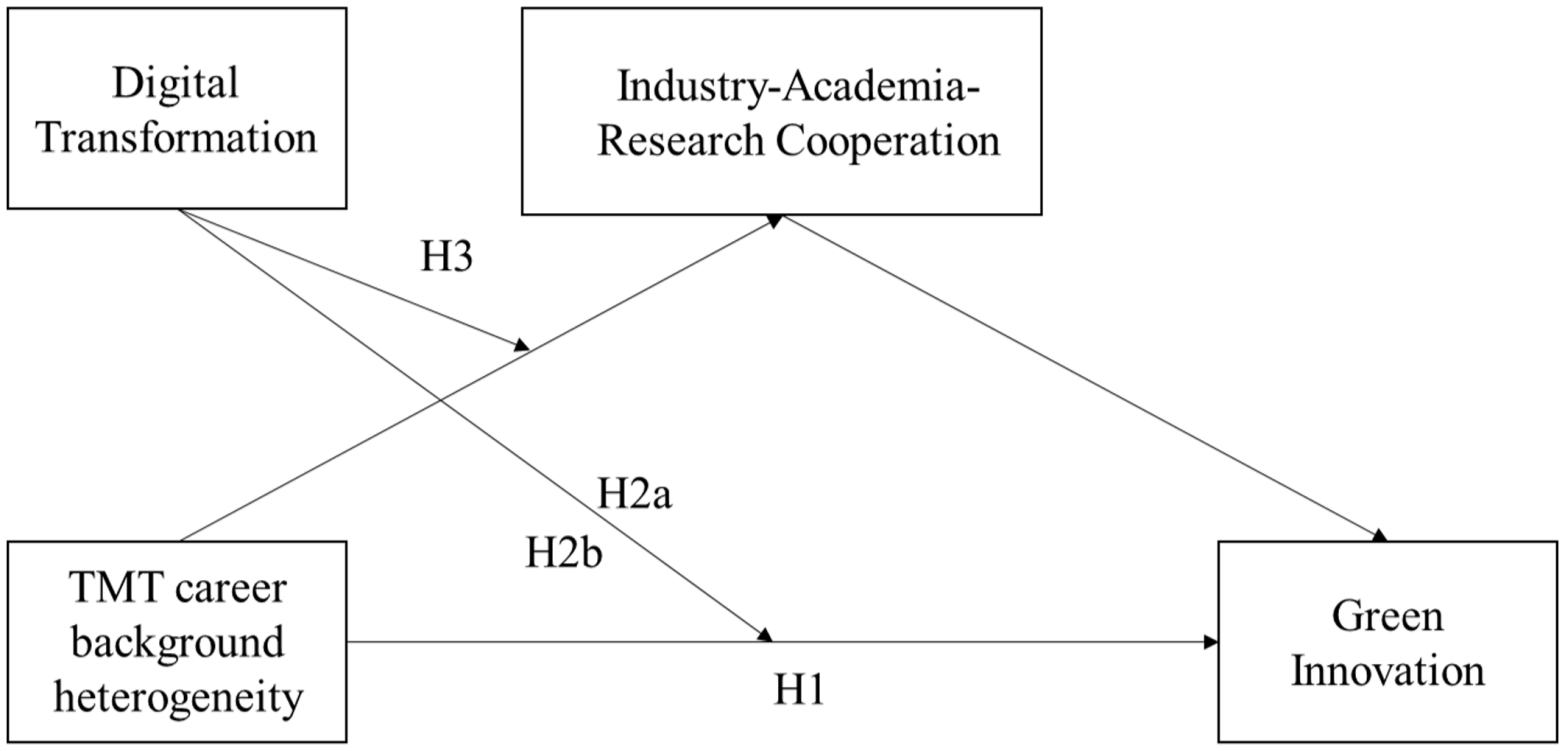
Theoretical framework: moderated mediation model.

The RBV offers a theoretical basis for treating digital transformation as a moderator variable rather than as a mediator in this study. Digital transformation directly impacts the relationship between unique resources, such as TMT heterogeneity, industry-academia-research cooperation, and the achievement of green innovation (Barney et al., 2011). Concurrently, the role of industry-academia-research cooperation as a mediator is underlined as it bridges the gap between these unique resources and green innovation (Zahra and Nambisan, 2012; Xu et al., 2022). Hence, leveraging the RBV, this study elaborates on the mediating role of industry-academia-research cooperation on the TMT career experience heterogeneity-green innovation relationship and the moderating role of corporate digital transformation on the mediating effect of industry-university-research cooperation, which will contribute to green innovation research. The theoretical framework is illustrated in Figure 1.

### 2.3. Top management team career experience heterogeneity and corporate green innovation

Career experience heterogeneity within top management teams (TMTs) is critical for strategic decision-making and performance, as suggested by upper echelons theory (Hambrick, 2007). TMTs comprising executives with varied career histories possess diverse perspectives, knowledge bases, and approaches that allow comprehensive analysis of problems from multiple angles (Nielsen, 2010; Buyl et al., 2011). For instance, executives with engineering backgrounds emphasize technical feasibility and pragmatic considerations (Garcia-Blandon et al., 2019), while those from marketing prioritize customer perspectives and conceptual visions (Janani et al., 2022). Integrating these diverse views enables optimized solutions that balance practical and creative considerations, leading to innovation (Certo et al., 2006; Cannella et al., 2008).

Furthermore, experience spanning different industries provides valuable insights into emerging innovation opportunities and challenges (Attah-Boakye et al., 2021). Executives who have worked in sustainability-adjacent domains such as renewable energy, waste management, and environmental compliance bring critical knowledge of green technologies, regulations, and stakeholder pressures (Amore et al., 2019). Their expertise helps identify impending trends, customer needs, and high-potential product directions to guide effective environmental strategies and green innovation (Kim et al., 2017).

The varied cognitive toolkits sparked by heterogeneous career experiences enhance team creativity as well (Crossland et al., 2014). Exposure to diverse thinking styles, problem-solving approaches, and creative techniques augments the repertoire of strategies that TMTs can employ for ideation and innovation (Harrison and Klein, 2007). Rather than conformity breeding groupthink, career experience diversity fosters novel recombination and synergistic integration of insights across domains to generate innovative solutions (Leroy et al., 2022). Therefore, we hypothesize:

*Hypothesis* 1: Career experience heterogeneity among corporate top management teams is positively associated with corporate green innovation.

### 2.4. The moderating role of corporate digital transformation

Digital transformation within the corporate environment can significantly influence the relationship between the heterogeneity of Top Management Team (TMT) career experiences and the propensity for green innovation. This relationship can be divided into two perspectives: data-driven and resource curse.

The data-driven perspective conceptualizes corporate digital transformation as the use of vast data and advanced analytics to strengthen decision-making, optimize operations, and spur innovation (Muntanyola-Saura, 2016; Nambisan et al., 2017). This transformation profoundly impacts organizational strategies and practices, including those related to green innovation (Yoo et al., 2010; George et al., 2014). The positive moderating effect of data-driven digital transformation on the TMT heterogeneity-green innovation relationship is attributable to several interrelated mechanisms. First, TMTs with diverse career experiences bring varied perspectives for analyzing problems and generating solutions (Tay and Loh, 2021). When complemented by data-driven insights, this enhances decision-making capabilities to devise innovative green strategies (Nguyen and Hoai, 2022). Second, advanced analytics uncover novel green innovation opportunities undetectable through traditional methods (Gunasekaran et al., 2017; Akter et al., 2020). Such data-driven opportunities allow diverse TMTs to apply their unique expertise for customized eco-innovation approaches (Kiron et al., 2014; Shen, 2017). Third, data-driven decision-making rapidly detects potential implementation risks and challenges, facilitating superior risk management (Segarra and Toledano, 2020). By elucidating these mechanisms, this discussion theoretically substantiates the positive moderating role of data-driven digital transformation in enabling diverse TMTs to realize green innovation. From a data-driven perspective, we propose the following hypothesis:

*Hypothesis* 2a: Corporate digital transformation positively moderates the impact of TMT career experience heterogeneity on green innovation.

On the other hand, the resource curse perspective suggests that while digital transformation appears beneficial, it can lead to negative effects due to an overabundance of resources, such as technology and data (Ahuja and Morris Lampert, 2001). These effects could include complacency, inefficiencies, and decreased focus on green innovation (Autio et al., 2013; Nambisan and Luo, 2021).

Several mechanisms can explain the negative moderating effect of corporate digital transformation on the relationship between TMT career experience heterogeneity and green innovation. First, companies may become too reliant on data, which could suffocate creativity and intuition, vital components of innovation (Bolton et al., 2015). This could be particularly pronounced in diverse management teams, where data might be used to suppress differing opinions rather than fostering innovative ideas (Shen, 2017). Second, rich resources can lead to misallocation or misuse (Huang and Meng, 2023). In the context of digital transformation, this could entail overinvestment in certain technologies at the expense of others such as green technologies (Aral and Weill, 2007; Ross et al., 2017). Third, the abundance of resources might breed complacency, leading companies to believe that they have everything they need to succeed (Tripsas and Gavetti, 2000; Christensen et al., 2016). This can generate resistance to change and innovation, especially in diverse teams, where change can be more challenging to implement (Bower and Christensen, 1995; Eggers and Kaplan, 2013). From the resource curse perspective, we propose the following hypothesis:

*Hypothesis* 2b: Corporate digital transformation negatively moderates the impact of TMT career experience heterogeneity on green innovation.

### 2.5. The moderating role of corporate digital transformation on the mediating effect of industry-university-research collaboration

Collaborations among industry, academia, and research institutions play an instrumental role in advancing green innovation (Siegel et al., 2004). By bridging knowledge gaps, they expedite the transfer and implementation of green technologies, a phenomenon well-documented in the literature (Perkmann et al., 2013). The heterogeneous backgrounds of top management teams (TMTs) in firms can foster diverse perspectives and ideas, which is a vital ingredient for green innovation (Hambrick, 2007). However, the translation of these ideas into tangible innovation often requires a mediating mechanism such as industry-academia-research collaboration (Powell et al., 1996).

In the era of digital transformation, the role of technology in strengthening these collaborations is becoming increasingly evident (Evans and Miklosik, 2023). By enhancing communication and coordination between a company and its external partners, digital transformation can bolster the mediating effect of industry-university-research collaboration on the TMT career experience in a heterogeneity-green innovation relationship (Bharadwaj et al., 2013; Nambisan et al., 2017). Specifically, digital transformation can provide effective platforms for collaboration, facilitate the sharing and integration of knowledge and resources, and enable real-time communication (Dionisio et al., 2023). This digital enablement strengthens collaboration among industry, academia, and research institutions and allows firms to efficiently process and integrate the acquired knowledge from these collaborations into their innovation processes (Rocha et al., 2022).

*Hypothesis* 3: Corporate digital transformation moderates the mediating effect of industry-university-research cooperation on TMT career experience in a heterogeneity-green innovation relationship. Specifically, the mediating effect of industry-university-research cooperation is more pronounced in the high degree of corporate digital transformation.

## 3. Data and research methodology

### 3.1. Sample selection and data sources

As the world’s largest developing economy and carbon emitter, China presents a critical focus for examining green innovation and sustainability transitions (Ahmad et al., 2018; Guo et al., 2018). The nation’s ecological development priorities are substantiated through substantial investments, policy reforms, and emission reduction targets (Zhang et al., 2018; Peng, 2020). With goals to achieve carbon neutrality by 2060 and peak emissions by 2030, China’s commitment to environmental issues is unequivocal (Xu et al., 2021). Green innovation has become a strategic focus for businesses and government alike as a pathway to meet climate goals (Song and Yu, 2018; Fu et al., 2022). Moreover, China’s rapid digital transformation provides an opportune context for exploring the intersection of technology, innovation, and sustainability (Zhao et al., 2008; Zhai et al., 2022). As the largest modernizing economy pursuing an ecological civilization, China’s sustainability experience offers transferable lessons for other emerging economies on leveraging digital capabilities for eco-innovation (Lin et al., 2014). Consequently, this study aims to provide timely empirical insights into green innovation dynamics in a major economy undertaking profound digital and sustainability transitions.

This research employs an unbalanced panel dataset, encompassing annual data from 19,155 A-share companies listed on the main boards of Shanghai and Shenzhen. The dataset strategically excludes special treatment (ST) firms, financial institutions, and companies with incomplete data, ensuring a concentrated and comprehensive examination of the most pertinent entities. The primary source of firm-level data is the WIND database, a comprehensive resource that emphasizes Chinese finance and economics. This selection process offers a wealth of reliable information. For patent data, the study turns to the Patent Statistics Office, adopting a categorization aligned with the World Intellectual Property Organization (WIPO) Green Innovation Basis. This classification system provides a universally accepted framework for identifying green patents. Additionally, the study extracts data on firms’ digital transformation from their annual reports and management discussion and analysis (MD&A). This approach enables this study to recognize and analyze the strategic integration of digital technologies within business operations and innovation efforts.

### 3.2. Variables

#### 3.2.1. Measure of top management team career experience heterogeneity

The Herfindahl–Hirschman Index (HHI) is commonly used to measure categorical diversity such as career experience heterogeneity within teams (Carpenter et al., 2004; Cannella et al., 2008; Wei and Wu, 2013). The HHI is calculated as follows:


$$\mathrm{hcareer}=\left(1-\sum \alpha_{i}^{2}\right)$$

Where $\alpha_{i}$ indicates the proportion of team members in category i relative to the total number of team members. The hcareer values range from 0 to 1, with higher values denoting greater heterogeneity (O'Reilly et al., 1989; Sheng et al., 2011).

#### 3.2.2. Measure of digital transformation

This study utilizes text mining and analysis techniques to systematically measure corporate digital transformation (Feliciano-Cestero et al., 2023). Specifically, we employ Python for web scraping and natural language processing of unstructured data from corporate disclosures (Saggion and Poibeau, 2013; Gandomi and Haider, 2015). The textual data are analyzed using the Java PDFBox library and other NLP tools to extract keywords related to digital transformation, based on an extensive dictionary compiled from seminal literature and policy documents (Chanias et al., 2019; Matt et al., 2023). These keywords are matched against the corpus of texts to derive frequency counts, a validated approach in management research (Rha and Lee, 2022). The resultant keyword frequencies provide a robust quantitative measure of digital transformation for each firm. This enables leveraging big data analytics to glean strategic insights from unstructured textual disclosures, enhancing methodological rigor (Tambe, 2014; Mithas et al., 2022).

**Figure 2:**
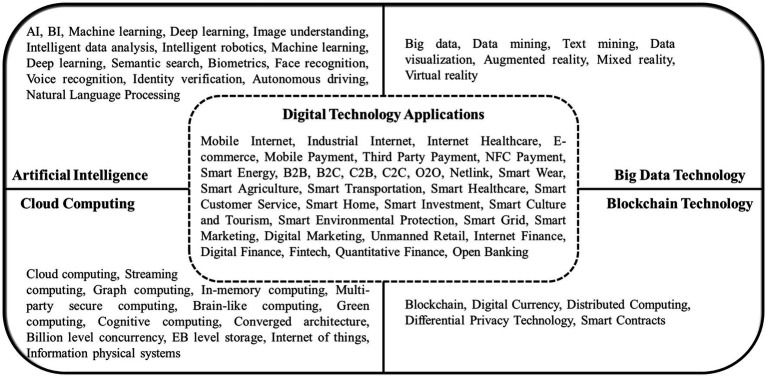
Corporate digital transformation keyword map.

In this study, we compile and organize the annual reports (Ren and Li, 2022; Shang et al., 2023) and management discussion and analysis (MD&A; Luo et al., 2023; Tu and He, 2023) of all A-share listed companies in Shanghai and Shenzhen stock exchanges to obtain the variables. This study references policy documents and industry reports to expand the inventory of digital transformation keywords (Annarelli et al., 2021; Nambisan and Luo, 2021). Resources such as the “Special Action Plan for Digital Empowerment of SMEs,” the “Implementation Plan for Promoting the ‘Cloud Usage for Intelligent Development’ Action to Nurture New Economic Development,” the “2020 Digital Transformation Trend Report,” and “Government Work Reports.” The keywords were subsequently structured into two categories: “Underlying Technology Application” and “Technology Practice Application,” as depicted in the keyword map in Figure 2 (Chan et al., 2006; Hess et al., 2020).

#### 3.2.3. Measure of control variable

To improve the accuracy of our results, we controlled for factors that may impact firm performance based on established studies. Specifically, we included corporate governance and profitability indicators that are associated with innovation outcomes (Lee and O'neill, 2003; Laursen and Salter, 2014). Governance measures comprise ownership concentration (Zheng C. et al., 2022), institutional ownership (García-Sánchez et al., 2020), and board size (Van Essen et al., 2015), which affect governance quality and innovation strategy. Profitability metrics, measured by return on assets (ROA; Li et al., 2018) and the EBIT ratio (Meles et al., 2023), account for available resources enabling innovation. The variables used in this study are shown in Table 2.

**Table 2:** The variables used in the study.

Variable code	Variable explanation
Explained variable	
ISUM	The number of green invention patents granted
TSUM	The number of green patents granted
Explanatory variables	
hcareer	Corporate top management team career experience heterogeneity
Mediation variables	
CC	Corporate has granted patents which are jointly applied with other corporate or institution
Moderating variables	
DTAMDA	Digital transformation of enterprises based on MD&A
DTAAR	Digital transformation of enterprises based on annual reports
Control variables	
top	Percentage of shares held by the top ten shareholders
inst	Percentage of institutional ownership
tat	Total asset turnover ratio
lnta	Corporate size
ebitr	The ratio of EBIT to total assets
roa	Return on assets

### 3.3. Research methodology

In our study, we used a Moderated Mediation Model via grouped regression to investigate our research hypotheses. We apply stepwise regression method of Baron and Kenny (1986) to examine both the mediating and moderating effects.

To test the influence of top management team career hetergeinty on corporate green innovation and the moderating effect of corporate digital transframation, i.e., Hypotheses 1 and 2, this study uses a panel data fixed effects model with the introduction of industry and time fixed effects (Fang et al., 2018).


$$Y_{i,t}=b_{0}+b_{1}X_{i,t}+e_{1}$$

$$Y_{i,t}=b_{0}+b_{2}M_{i,t}+e_{2}$$

$$Y_{i,t}=b_{0}+b_{1}X+b_{2}M_{i,t}+b_{3}X_{i,t}*M_{i,t}+e_{3}$$

where $Y_{i,t}$ is the explained variable for firm i at time t . $X_{i,t}$ is the explanatory variable for firm i at time t . $M_{i,t}$ is the moderating variable for firm i at time t .

To test the moderated mediation model (Hypothesis 3), we conducted subgroup analysis using moderated regression procedures (Baron and Kenny, 1986; Edwards and Lambert, 2007). Specifically, we categorized the sample into high and low digital transformation groups based on the median split of the digital transformation index. We then conducted mediation tests in each subgroup separately and estimated the following regression models in the high-digital-transformation subgroup:


$$Y_{i,t}=c_{1}X_{i,t}+e_{4}$$

$$M_{i,t}=a_{1}X_{i,t}+e_{5}$$

$$Y_{i,t}={c_{1}}^{\prime }X+b_{1}M_{i,t}+e_{6}$$

Where $M_{i,t}$ is the mediating variable, $X_{i,t}$ is the explanatory variable, and $Y_{i,t}$ is the explanatory variable. $a_{1}$ represents the effect of X on M , $b_{1}$ represents the effect of M on Y , and ${c_{1}}^{\prime }$ is the direct effect of X on Y after controlling for M .

Similarly, in the low digital transformation subgroup, we estimated:


$$Y_{i,t}=c_{1}X_{i,t}+e_{4}$$

$$M_{i,t}=a_{1}X_{i,t}+e_{5}$$

$$Y_{i,t}={c_{1}}^{\prime }X+b_{1}M_{i,t}+e_{6}$$

We then compared the regression coefficients $a_{1}$ vs. $a_{2}$ and $b_{1}$ vs. $b_{2}$ between the two subgroups. If the effects differ significantly across subgroups, it indicates a moderated mediation effect, where digital transformation moderates the indirect effect of X on Y through M .

## 4. Results

### 4.1. Descriptive statistics

Tables 3, 4 report the descriptive statistics and correlations of the sample. These variables are based on an unbalanced panel dataset of 19,155 Chinese A-share firm-year observations from 2011 to 2020. The descriptive statistics in Table 2 show that, on average, firms generate 2.08 green patents (TSUM) and 0.55 green invention patents (ISUM). There is also notable variation in top management team (TMT) career experience heterogeneity (hcareer; mean = 0.66) and digital transformation measured through corporate announcements (DTAMDA; mean = 3.90) and annual reports (DTAAR; mean = 8.64). The correlation matrix in Table 3 shows that the correlations between variables were mostly modest. TMT career experience diversity (hcareer) has small positive correlations with green patents (TSUM; *r* = 0.055) and inventions (ISUM; *r* = 0.062). Digital transformation (DTAMDA and DTAAR) also correlates with innovation outcome. Importantly, multicollinearity was not a concern, as the correlations among predictors were below 0.50.

**Table 3:** Descriptive statistics on key variables.

Variable	(1)	(2)	(3)	(4)	(5)	(6)
	*N*	Mean	Median	STD	Min	Max
TSUM	19,155	2.080	0	9.650	0	186
ISUM	19,155	0.550	0	2.860	0	53
hcareer	19,155	0.660	0.690	0.110	0	1
CC	19,155	0.360	0	0.480	0	1
DTAMDA	19,155	3.900	0	10.54	0	209
DTAAR	19,155	8.640	1	23.10	0	467

**Table 4:** Correlation coefficients.

	TSUM	ISUM	hcareer	CC	DTAMDA	DTAAR
TSUM	1					
ISUM	0.826^***^	1				
hcareer	0.055^***^	0.062^***^	1			
CC	0.172^***^	0.173^***^	0.076^***^	1		
DTAMDA	0.042^***^	0.071^***^	0.006	0.062^***^	1	
DTAAR	0.060^***^	0.092^***^	0.008	0.060^***^	0.865^***^	1

### 4.2. Empirical results

#### 4.2.1. Baseline regression results

**Figure 3:**
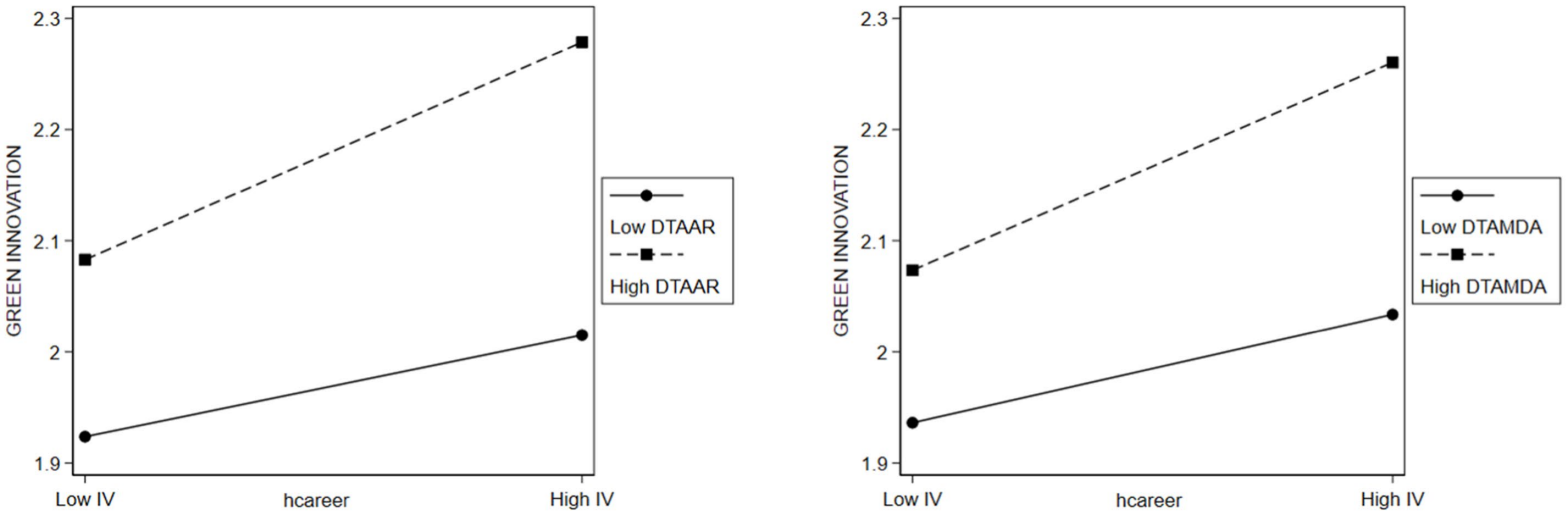
Moderating role of corporate digital transformation.

Table 5 shows the regression results of the base regression of corporate top management team career experience heterogeneity with corporate green innovation and the moderating effect of corporate digital transformation. Our research findings underscore the benefits of Top Management Team (TMT) career experience heterogeneity on green innovation (β=2.067,p<0.01 ), corroborating the existing literature that highlights the innovation impact of TMT heterogeneity (Xie et al., 2022). In line with upper echelons theory, our results suggest that diverse career histories within TMTs can foster creativity and stimulate green innovation by bringing unique viewpoints and knowledge about environmental technologies and strategies (Hambrick, 2007). We further discovered a positive correlation between digital transformation and green innovation ( β=0.028,p<0.01;β=0.015,p<0.01 ), which is consistent with pervious findings (Xue et al., 2022; Sun and He, 2023). In addition, corporate digital transformation has a positive moderating effect on the relationship between TMT career experience heterogeneity and green innovation ( β=0.210,p<0.01;β=0.112,p<0.01 ). A schematic representation of the moderating effect is shown in Figure 3. This suggests that digital transformation can more effectively leverage TMT career experience heterogeneity for green innovation, reinforcing the argument that a variety of knowledge and perspectives–a byproduct of diversity–is essential for harnessing new technologies (Vial, 2019).

**Table 5:** Panel OLS regression of the moderating effects of corporate digital transformation.

	(1)	(2)	(3)	(4)	(5)
	TSUM	TSUM	TSUM	TSUM	TSUM
hcareer	2.067^***^		1.291^**^		1.164^*^
	(3.512)		(2.086)		(1.877)
DTAMDA		0.028^***^	−0.109^***^		
		(3.911)	(−3.092)		
DTAMDA*hcareer			0.210^***^		
			(3.963)		
DTAAR				0.015^***^	−0.058^***^
				(4.801)	(−3.492)
DTAAR*hcareer					0.112^***^
					(4.500)
_cons	−43.032^***^	−41.571^***^	−42.278^***^	−41.390^***^	−42.014^***^
	(−32.635)	(−32.581)	(−31.908)	(−32.405)	(−31.673)
Control	Yes	Yes	Yes	Yes	Yes
Industry	Yes	Yes	Yes	Yes	Yes
Year	Yes	Yes	Yes	Yes	Yes
*N*	19,155	19,155	19,155	19,155	19,155
*r* ^2^	0.154	0.154	0.155	0.154	0.155

*t* statistics are in parentheses; ^*^p < 0.1, ^**^p < 0.05, ^***^p < 0.01.

#### 4.2.2. Moderated mediation analysis

Table 6 presents the results of tests for the moderate mediation model. The results indicate a significant moderated mediation effect, whereby the indirect effect of TMT career experience heterogeneity (hcareer) on green innovation through industry-academia-research cooperation (CC) is contingent on the level of digital transformation. Specifically, the mediating effect of CC was stronger when digital transformation was high ( a1=0.127,p<0.01;b1=1.955,p<0.01 ) than it was when it was low ( a2=0.090,p<0.01;b2=1.072,p<0.01 ). This supports Hypothesis 3 that corporate digital transformation positively moderates the mediating effect of CC on the relationship between TMT heterogeneity and green innovation (Hayes, 2017). In addition, the mediating effect of industry-academia-research cooperation (CC) in the high digital transformation group is significant, validating the theorized mechanism by which corporate digital transformation enhances the mediating role of industry-academia-research cooperation in translating TMT heterogeneity into greater green innovation (Preacher et al., 2007). This highlights the enabling effect of digital transformation in leveraging collaborative capabilities to increase eco-innovation.

**Table 6:** Moderated mediation analysis.

	(1)	(2)	(3)	(4)	(5)	(6)
	High Digital Transformation Group			Low Digital Transformation Group		
	TSUM	CC	TSUM	TSUM	CC	TSUM
hcareer	3.889^***^ (2.985)	0.127^**^ (2.535)	3.640^***^ (2.800)	0.949 (1.633)	0.090^***^ (2.608)	0.852 (1.469)

CC			1.955^***^ (6.122)			1.072^***^ (7.171)
				
top	0.004 (0.357)	−0.003^***^ (−7.997)	0.009 (0.958)	0.010^**^ (2.362)	−0.002^***^ (−6.274)	0.012^***^ (2.765)
inst	−0.022^***^ (−3.163)	0.001^***^ (4.338)	−0.024^***^ (−3.494)	−0.002 (−0.605)	0.000 (1.085)	−0.002 (−0.676)

tat	−0.231 (−0.707)	−0.009 (−0.678)	−0.215 (−0.657)	0.170 (1.118)	0.021^**^ (2.303)	0.148 (0.973)

lnta	3.087^***^ (23.987)	0.131^***^ (26.328)	2.832^***^ (20.982)	1.366^***^ (23.366)	0.144^***^ (41.204)	1.212^***^ (19.495)

ebitr	−5.129 (−0.931)	−0.618^***^ (−2.908)	−3.921 (−0.713)	−4.136^*^ (−1.739)	−0.053 (−0.375)	−4.079^*^ (−1.719)

roa	−0.003 (−0.069)	0.006^***^ (3.309)	−0.014 (−0.319)	0.019 (0.961)	0.001 (1.147)	0.017 (0.889)

_cons	−67.927^***^ (−23.223)	−2.493^***^ (−22.110)	−63.051^***^ (−20.853)	−30.127^***^ (−22.954)	−2.862^***^ (−36.578)	−27.058^***^ (−19.636)

Industry	Yes	Yes	Yes	Yes	Yes	Yes
Year	Yes	Yes	Yes	Yes	Yes	Yes
*N*	6,590	6,590	6,590	12,565	12,565	12,565
*r* ^2^	0.183	0.217	0.188	0.143	0.184	0.146

*t* statistics are in parentheses; ^*^p < 0.1, ^**^p < 0.05, ^***^p < 0.01.

### 4.3. Heterogeneity analysis

State-owned enterprises (SOEs) have historically played a pivotal role in China’s economic development and growth (Lin et al., 2020). Understanding the differences between SOEs and non-SOEs is important, given their distinct institutional contexts and strategic priorities (Jones and Zou, 2017; Lin et al., 2020). In addition, the Yangtze River Digital Economic Belt initiative promotes China’s digital transformation and economic upgrading, especially in the Yangtze region (Wang et al., 2023). Examining the differences between firms located inside and outside this region provides insights into China’s regional digital divide and disparities in digital infrastructure.

Table 7 shows the heterogeneity analysis of SOEs and non-SOEs. Table 8 shows the heterogeneity analysis of enterprises located in the Yangtze River Economic Belt and the Non-Yangtze River Economic Belt. In addition, to further analyze the effect of digital transformation and TMT career experience heterogeneity on corporate green innovation, we used Fisher’s permutation test to test the coefficient differences between SOEs and non-SOEs, and the Yangtze River Economic Belt and Non-Yangtze River Economic Belt (Berry et al., 2002). The results of the Fisher’s permutation test are shown in Tables 9, 10. The results show that TMT career experience heterogeneity (hcareer) has a stronger positive effect on green innovation in non-SOEs than in SOEs. This aligns with research showing that non-SOEs have greater flexibility and market incentives, allowing TMT heterogeneity to stimulate innovation (Luo et al., 2010). Both the moderating effect and direct effect of digital transformation of SOEs are higher than those of non-SOEs, which emphasizes the importance of digital transformation for state-owned enterprises (Nambisan et al., 2017). Regarding regions, both the moderating and direct effects of digital transformation were stronger in the non-Yangtze River Economic Belt than in the Yangtze River Economic Belt. This illustrates the equal importance of corporate digitization in the non-Yangtze River Economic Belt.

**Table 7:** Heterogeneity analysis: SOEs and non-SOEs.

	(1)	(2)	(3)	(4)	(5)	(6)
	State-owned enterprises			Non-State-owned enterprises		
	TSUM	TSUM	TSUM	TSUM	TSUM	TSUM
hcareer	1.189 (1.305)		0.110 (0.113)	2.884^***^ (3.787)		2.162^***^ (2.693)
		
DTAMDA		0.033^**^ (2.323)	−0.246^***^ (−2.605)		0.025^***^ (3.113)	−0.077^**^ (−2.151)
		
DTAMDA *hcareer			0.404^***^ (2.980)			0.160^***^ (2.925)
			
_cons	−45.241^***^ (−22.398)	−44.382^***^ (−22.675)	−44.513^***^ (−21.952)	−42.595^***^ (−23.420)	−40.415^***^ (−22.861)	−41.631^***^ (−22.733)

Control	Yes	Yes	Yes	Yes	Yes	Yes
Industry	Yes	Yes	Yes	Yes	Yes	Yes
Year	Yes	Yes	Yes	Yes	Yes	Yes
*N*	8,709	8,709	8,709	10,446	10,446	10,446
*r* ^2^	0.175	0.176	0.177	0.139	0.139	0.141

*t* statistics are in parentheses; ^*^p < 0.1, ^**^p < 0.05, ^***^p < 0.01.

**Table 8:** Test for differences between groups: SOEs and Non-SOEs.

	(1)	(2)	(3)
	Coefficient: SOEs	Coefficient: Non-SOEs	Empirical *p* value
	TSUM	TSUM	TSUM
hcareer	1.189	2.884^***^	−1.695^***^
DTAMDA	0.033^**^	0.025^***^	0.008^**^
DTAMDA*hcareer	0.404^***^	0.160^***^	0.244^***^

Empirical *p* values were used to test the significance of the differences in regression coefficients between the SOE and non-SOE in Table 7, and empirical *p* values were obtained by bootstrap sampling 1,000 times based on Fisher’s permutation test. ^*^p < 0.1, ^**^p < 0.05, and ^***^p < 0.01.

**Table 9:** Heterogeneity analysis: Yangtze River Economic Belt and Non-Yangtze River Economic Belt.

	(1)	(2)	(3)	(4)	(5)	(6)
	Yangtze River Economic Belt			Non-Yangtze River Economic Belt		
	TSUM	TSUM	TSUM	TSUM	TSUM	TSUM
hcareer	1.793^***^ (2.689)		1.170 (1.617)	2.221^**^ (2.359)		1.404
						(1.436)
DTAMDA		0.023^**^ (2.308)	−0.087^*^ (−1.679)		0.031^***^ (3.056)	−0.115^**^ (−2.381)
		
DTAMDA *hcareer			0.167^**^ (2.152)			0.223^***^ (3.094)
			
_cons	−30.827^***^ (−19.506)	−29.589^***^ (−19.263)	−30.224^***^ (−18.975)	−51.305^***^ (−25.325)	−49.737^***^ (−25.480)	−50.528^***^ (−24.859)

Control	Yes	Yes	Yes	Yes	Yes	Yes
Industry	Yes	Yes	Yes	Yes	Yes	Yes
Year	Yes	Yes	Yes	Yes	Yes	Yes
*N*	8,991	8,991	8,991	10,164	10,164	10,164
*r* ^2^	0.150	0.150	0.151	0.163	0.163	0.165

*t* statistics are in parentheses; ^*^p < 0.1, ^**^p < 0.05, ^***^p < 0.01.

**Table 10:** Test for differences between groups: Yangtze River Economic Belt and Non-Yangtze River Economic Belt.

	(1)	(2)	(3)
	Coefficient	Coefficient:	Empirical *p* value
	Yangtze River Economic Belt	Non-Yangtze River Economic Belt	
	TSUM	TSUM	TSUM
hcareer	1.793^***^	2.221^**^	0.428^**^
DTAMDA	0.023^**^	0.031^***^	−0.008^**^
DTAMDA*hcareer	0.167^***^	0.223^***^	−0.057 ^*^

Empirical *p* values were used to test the significance of the differences in regression coefficients between the Yangtze River Economic Belt and non-Non-Yangtze River Economic Belt in Tables 9, and empirical *p* values were obtained by bootstrap sampling 1,000 times based on Fisher’s permutation test. ^*^p < 0.1, ^**^p < 0.05, ^***^p < 0.01.

### 4.4. Endogeneity issues

In this study, we employ various techniques to address potential endogeneity concerns. Specifically, we used the propensity score matching difference-in-differences (PSM-DID), two-stage least squares (2SLS), and lagged independent variable models. Each of these methods has unique strengths in addressing the different facets of endogeneity, and their collective use reinforces the robustness of our findings.

We utilized PSM-DID to deal with the problem of selection bias and control for time-invariant unobserved factors that may simultaneously influence Top Management Team (TMT) heterogeneity and innovation (Shipilov, 2009; Luo et al., 2010). Using this method, we distinguished between a control group and an experimental group based on whether a company is undergoing digital transformation. We used 2016 as the cutoff point in this analysis because of its significance in China’s “13th Five-Year Plan” for National Informatization, which emphasized the construction of “Digital China”(Daojiong and Dong, 2022). This marked a major shift in the country’s digital landscape, with the proliferation of digital applications, such as intelligent manufacturing, digital finance, digital government, and intelligent transportation. This approach allows us to isolate the effects of digital transformation on TMT heterogeneity and innovation. The results of the PSM-DID regression are presented in Table 11. We find that the coefficients of DID are all positive and significant.

**Table 11:** PSM-DID regression.

	(1)	(2)	(3)
	TSUM	TSUM	TSUM
DID	1.122^***^	1.005^***^	0.981^***^
	(5.540)	(4.826)	(4.709)
hcareer	1.922^***^		0.999
	(2.986)		(1.457)
DTAMDA		0.017^**^	−0.116^***^
		(2.356)	(−3.270)
DTAMDA*hcareer			0.203^***^
			(3.847)
_cons	−47.595^***^	−46.324^***^	−46.840^***^
	(−30.351)	(−30.307)	(−29.717)
Control	Yes	Yes	Yes
Industry	Yes	Yes	Yes
Year	Yes	Yes	Yes
N	15,705	15,705	15,705
r2	0.155	0.154	0.156

*t* statistics are in parentheses; ^*^p < 0.1, ^**^p < 0.05, ^***^p < 0.01.

To address the potential reverse causality, in which green innovation could influence TMT heterogeneity, we employed 2SLS (Antonakis et al., 2010). This method uses the industry average of TMT heterogeneity and level of provincial digital economy development as instruments for firm-level TMT heterogeneity and digital transformation, respectively. These instruments meet the relevance and exogeneity conditions (Bascle, 2008). The rationale behind choosing these instruments lies in their strong correlation with endogenous variables and their presumed lack of correlation with the error term. The results of the instrumental variable two-stage least squares regression regression are shown in Table 12. The coefficients of hcareer and DTAMDA remain significant in the second stage, with the first-stage F-statistic exceeding 10, indicating that these are strong instruments.

**Table 12:** Instrumental variable 2-stage least squares regression.

	(1)	(2)	(3)	(4)
	First stage	Second stage	First stage	Second stage
	hcareer	TSUM	DTAMDA	TSUM
hcareer_mean	−0.331^***^			
	(−4.064)			
hcareer		74.742^***^		
		(2.827)		
PDED			7.107^***^	
			(8.372)	
DTAMDA				0.834^***^
				(7.493)
_cons	0.713^***^	−81.486^***^	−14.387^***^	−30.206^***^
	(14.105)	(−5.914)	(−12.652)	(−12.429)
Control	Yes	Yes	Yes	Yes
Industry	Yes	Yes	Yes	Yes
Year	Yes	Yes	Yes	Yes
First-stage *F*-Value	17.02		132.55	
*N*	19,155	19,155	19,155	19,155
*r* ^2^	0.058	0.154.	0.284	0.0.157

*t* statistics are in parentheses; ^*^p < 0.1, ^**^p < 0.05, ^***^p < 0.01.

Finally, to account for autocorrelation and unobserved heterogeneity, we used lagged dependent variable models (Wooldridge, 2010). By lagging the dependent variable by 1 year, we could rule out simultaneity concerns. The results of the lagged independent variable regression are listed in Table 13, and the coefficients are consistent with those in Table 5. This approach suggests that the positive effects of TMT heterogeneity and digital transformation persist, thus reinforcing the validity of our findings.

**Table 13:** Lagged independent variable regression.

	(1)	(2)	(3)	(4)	(5)
	L.TSUM	L.TSUM	L.TSUM	L.TSUM	L.TSUM
hcareer	1.127^***^		0.772^***^		0.671^**^
	(4.246)		(2.752)		(2.386)
DTAMDA		0.013^***^	−0.043^***^		
		(4.253)	(−2.869)		
DTAMDA*hcareer			0.086^***^		
			(3.818)		
DTAAR				0.007^***^	−0.027^***^
				(4.904)	(−3.767)
DTAAR*hcareer					0.051^***^
					(4.809)
_cons	−19.951^***^	−19.167^***^	−19.597^***^	−19.090^***^	−19.455^***^
	(−33.703)	(−33.480)	(−32.932)	(−33.309)	(−32.660)
Control	Yes	Yes	Yes	Yes	Yes
Industry	Yes	Yes	Yes	Yes	Yes
Year	Yes	Yes	Yes	Yes	Yes
*N*	16,902	16,902	16,902	16,902	16,902
*r* ^2^	0.102	0.102	0.103	0.102	0.104

*t* statistics are in parentheses; ^*^p < 0.1, ^**^p < 0.05, ^***^p < 0.01.

In conclusion, these methodological choices enabled us to mitigate endogeneity concerns, including reverse causality, omitted variable bias, and simultaneity. The consistent positive effects across the different model specifications lend further credence to the hypothesized relationships between TMT heterogeneity, digital transformation, and innovation. By triangulating the results using these complementary methods, we provided robust evidence of causal relationships, augmenting the validity and reliability of our study.

### 4.5. Robustness checks

Some scholars have counted 99 digitization-related word frequencies in four dimensions: digital technology applications, Internet business models, smart manufacturing, and modern information systems, to represent the degree of digital transformation of enterprises (Liu et al., 2023; Nguyen-Thi-Huong et al., 2023). Therefore, we examine the robustness of the data using the new metrics as a proxy for firms’ digital transformation. In addition, some scholars have suggested that green innovation can be measured using the number of patents granted to inventions (Gao et al., 2023). The findings in Table 14 show that the coefficients are statistically significant, consistent with previous research.

**Table 14:** Robustness checks.

	(1)	(2)	(3)	(4)	(5)
	ISUM	ISUM	ISUM	ISUM	ISUM
hcareer	0.839^***^ (4.685)		0.347^*^ (1.713)		0.387^*^ (1.905)
		
DTBMDA		0.007^***^ (6.425)	−0.020^***^ (−3.723)		
			
DTBMDA*hcareer			0.042^***^ (5.119)		
				
DTBAR				0.004^***^ (8.106)	−0.007^***^ (−2.976)
			
DTBAR*hcareer					0.017^***^ (4.502)
				
_cons	−12.406^***^ (−30.922)	−11.741^***^ (−30.219)	−11.894^***^ (−29.251)	−11.675^***^ (−30.055)	−11.874^***^ (−29.229)

Control	Yes	Yes	Yes	Yes	Yes
Industry	Yes	Yes	Yes	Yes	Yes
Year	Yes	Yes	Yes	Yes	Yes
*N*	19,155	19,155	19,155	19,155	19,155
*r* ^2^	0.110	0.111	0.113	0.112	0.114

*t* statistics are in parentheses; ^*^p < 0.1, ^**^p < 0.05, ^***^p < 0.01.

## 5. Discussion

### 5.1. Theoretical contributions

First, this study addresses a notable gap in the upper echelons literature by examining the impact of top management team (TMT) career experience heterogeneity on corporate green innovation. While existing research has focused predominantly on individual executive traits, this study shifts its lens to collective team diversity, providing novel insights into how the configuration of TMTs shapes strategic outcomes. By illuminating the relationship between TMT career experience heterogeneity and green innovation propensity, this study expands and enriches upper echelons theory.

Second, this study integrates the resource-based view to explain the mechanisms underlying the link between TMT heterogeneity and green innovation. The introduction of digital transformation and industry-academia-research cooperation as moderators and mediators, respectively, provides a nuanced elaboration of how diverse TMTs translate ideas into impactful eco-innovations. This theoretical augmentation helps address the “black box” limitation of the upper echelons theory pertaining to the opaque inner workings of the strategy formation process. Overall, incorporating the resource-based perspective contributes to a value-adding theoretical dimension.

Third, this study expands the application of upper echelons theory to the increasingly critical domain of corporate green innovation. By leveraging this well-established theoretical framework to examine an emerging strategic priority, this study demonstrates the versatility and predictive validity of the upper echelons theory across contexts. Examining TMT heterogeneity in relation to an important contemporary phenomenon enhances the relevance and robustness of this predominant theory.

In conclusion, this study’s theoretical contributions include addressing gaps concerning team diversity effects, elucidating mediating mechanisms via an integrated perspective, and extending upper echelons theory’s boundaries by linking TMT heterogeneity to salient challenges. By advancing knowledge of the strategic role of TMTs in enabling sustainability, this study provides important theoretical insights.

### 5.2. Practical contributions

This research has several salient practical implications for managers aiming to strategically foster corporate green innovation. First, the findings highlight the innovation-enhancing benefits of cultivating diversity within top management team (TMT) career histories and perspectives. This suggests that increased attentiveness to compositional considerations during TMT formation may be advantageous. Second, proactive investment in digital transformation capabilities emerges as an impactful means of optimizing the translation of TMT heterogeneity into actionable eco-innovations. This implies the value of dedicating resources for progressive digital integration. Third, actively pursuing collaborative relationships with external academia and research partners is a vital pathway for leveraging the knowledge exchange essential to actualizing inventive ideas.

Additionally, heterogeneity analysis of organizational differences offers further pragmatically relevant insights. The greater agility exhibited by private enterprises in harnessing diverse TMTs to drive innovation underscores the need for state-owned firms to address the structural and contextual barriers. The direct and indirect effects of digital transformation on green innovation in SOEs are significantly higher than in non-SOEs, reflecting the importance of digital transformation for SOEs. Furthermore, regional disparities in digitalization proficiency point to the imperative for digitally disadvantaged areas to prioritize infrastructure advancement to benefit firms. Finally, despite the PSM-DID model, we find the validated effectiveness of China’s 2016 national digital transformation policies, suggesting the important role of a supportive regulatory environment in unlocking innovation opportunities.

This study provides actionable guidance for managers seeking to cultivate progressive governance practices and partnerships to unlock the strategic innovation-enabling potential of diverse leadership augmented by digital capabilities. The multiple practical contributions center on illuminating paths for firms to leverage TMT composition and digital integration to further green innovation.

### 5.3. Limitations and future research

This study has certain limitations, which present avenues for future research. First, it relies on secondary patent data that may not fully capture a firm’s green innovation output. Surveys and interviews provided richer insights. Second, the sample comprises of only Chinese listed firms. Testing the model in other institutional contexts would improve its generalizability. Third, additional team diversity dimensions beyond career experience should be examined. Finally, longitudinal studies tracking firms over longer periods could better ascertain causal relationships. Future research could utilize data from diverse economies to evaluate the robustness of findings based on digital maturity and IT application levels. Moreover, exploring how country-specific factors like regulatory regimes, cultural values, industry composition, and development stages influence the mechanisms linking TMT diversity and green innovation will enrich the literature. Adopting cross-country lenses and multi-level perspectives to uncover variations across digital and sustainability contexts will deepen theoretical insights and inform policies for global green transformation.

## 6. Conclusion

This study integrates upper echelons theory and the resource-based view to investigate how top management team (TMT) career experience heterogeneity and corporate digital transformation interact to impact green innovation. An analysis of a comprehensive panel of 19,155 Chinese listed firms from 2011 to 2020 yields several key conclusions. First, TMT career experience heterogeneity has a positive effect on green innovation, mediated through enhanced industry-academia-research cooperation. This highlights the benefits of diverse perspectives on knowledge-sharing, which are critical to innovation. Second, digital transformation strengthens this relationship by providing effective platforms for collaboration with external partners, as evidenced by its positive moderating effect. This underscores the amplifying role of technology in optimizing team diversity advantages. Third, non-SOEs show more agility than SOEs in leveraging heterogeneous TMT to drive green innovation, implying that public-sector firms need to make organizational changes. Conversely, green innovation in SOEs benefits more from digital transformation, which includes both its direct and indirect effects of digital transformation. Fourth, lagging digital infrastructure in less-developed regions constrains firms from fully utilizing digital capabilities for eco-innovation. This suggests that the state should focus more on the balanced development of the digital economy as well as the even distribution of digital resources. Fifth, the validated effectiveness of China’s 13th Five-Year National Informatization Plan in propelling digital transformation implies a significant impact of policy initiatives. This was further evidenced by the positive differences found in the propensity score-matching difference-in-differences model. This research addresses critical gaps in the upper echelon literature concerning team diversity and sustainability while enriching the theory through an integrated perspective. This study delivers actionable insights into how companies can leverage inclusive leadership and digital integration to reorient their strategic priorities toward ecological sustainability.

## Data availability statement

The original contributions presented in the study are included in the article/supplementary material, further inquiries can be directed to the corresponding author/s.

## Author contributions

DG: Conceptualization, Data curation, Formal analysis, Methodology, Writing – original draft. SL: Project administration, Writing – review & editing. CG: Conceptualization, Methodology, Writing – original draft.
